# Redesigning miR-34a: structural and chemical advances in the therapeutic development of an miRNA anti-cancer agent

**DOI:** 10.1042/BST20253010

**Published:** 2025-08-04

**Authors:** Shreyas G. Iyer, Ikjot S. Sohal, Andrea L. Kasinski

**Affiliations:** 1 Department of Biological Sciences, Purdue University, West Lafayette IN, 47907, U.S.A.; 2 Purdue University Life Sciences Graduate Program, Purdue University, West Lafayette IN, 47907, U.S.A.; 3 Purdue Institute for Cancer Research, Purdue University, West Lafayette IN, 47907, U.S.A.

**Keywords:** microRNA, gene therapy, folate, cancer, miR-34

## Abstract

MicroRNAs (miRNAs) represent a promising class of therapeutics due to their ability to down-regulate multiple genes simultaneously. This offers a significant therapeutic advantage in cancer, where heterogeneity often activates different pathways in different patients. Chemical modifications to the miRNA help overcome challenges associated with nuclease susceptibility, high immunogenicity, and the need for high or repeated dosing to achieve therapeutic effects. The main chemical modifications include changes to the ribose and backbone. Ribose modifications, including 2′-O-methyl and 2′-fluoro, improve nuclease resistance and plasma stability and lower the immunogenicity of the miRNA. Phosphorothioate (PS) backbone modifications increase resistance to nucleases and prolong circulation by enhancing serum protein affinity. Integrating these stabilizing chemical modifications with ligand targeting allows for specific delivery of the chemically modified miRNAs to tumors and metastases, bypassing bulky delivery vehicles and improving penetration into dense tumor architectures. Enhancements to ligand chemistry can also overcome endosomal entrapment. Incorporating many of the modifications discussed in this mini-review, the first fully modified version of miR-34a (FM-miR-34a) was developed, marking a significant milestone as the first fully modified miRNA to demonstrate substantial in vivo activity. Ongoing optimization of the chemical modifications and ligand chemistry, and integrating artificial intelligence into the design process are expected to further extend the potential for delivering on the promise of using these Nobel Prize-winning miRNAs as anti-cancer agents.

## MicroRNA-34a, a small RNA with therapeutic promise

MicroRNAs (miRNAs) are short noncoding RNAs capable of down-regulating multiple genes simultaneously, exhibiting distinct expression patterns across various tissues and developmental stages [1–3]. This multi-targeting capability offers significant therapeutic advantages over siRNA, small molecules, or antibody-based treatments, which typically focus on one molecular target. Down-regulating multiple targets simultaneously allows an miRNA to influence the expression of multiple genes involved in related cellular processes and pathways, amplifying the cellular response and making them powerful therapeutics for restoring cellular functions disrupted by disease. This broad targeting ability is particularly valuable in cancer, where heterogeneity often leads to activation of multiple and varying pathways in different patients. Consequently, miRNA-based therapies may provide a more effective treatment across a diverse patient population [4].

Early pre-clinical studies involved injection of miRNA mimics either systemically or locally at target tissue sites, without the use of a delivery vehicle or without being encoded from viral vectors [5]. However, these approaches encountered challenges such as degradation of the miRNA in the bloodstream and inefficient delivery of systemically administered miRNA mimics to the target site, as well as difficulties with local delivery [6]. As a result, these methods achieved limited success in clinical translation. The development of advanced RNA chemistries and delivery technologies, including nanoparticle systems, has since enabled the first miRNA-based agents to enter clinical trials [7].

Among the miRNAs dysregulated in cancer, the miR-34 family—which includes miR-34a, miR-34b, and miR-34c and miR-449a, miR-449b, and miR-449c—has garnered significant attention due to down-regulation of the family members in lung, breast, and several other cancers [8,9]. The loss of these miRNAs can be attributed in part to their direct (miR-34) or indirect (miR-449) regulation by the tumor suppressor p53, which is mutated in over 50% of cancers [10,11]. The most extensively studied family member, miR-34a, represses an mRNA network that includes several oncogenic pathways involved in cell proliferation, migration, invasion, resistance to apoptosis, and immune evasion [12,13]. Key targets include the androgen receptor (AR), C-MYC, AXL, MET, SIRT1, CD44, PDL-1, as well as genes that influence the expression of cell cycle regulators such as cyclin-dependent kinase 4 (CDK4) and 6 (CDK6), and anti-apoptotic proteins like BCL-2 [14–24]. Evidently, miR-34a regulates a broad spectrum of clinically relevant targets, many of which are already the focus of small-molecule inhibitors and approved therapeutics [25–28]. This extensive reach allows miR-34a to function akin to a multi-drug cocktail, simultaneously modulating key oncogenic pathways to enhance therapeutic efficacy. Additionally, miR-34a functions as both a prognostic biomarker and a regulator of chemotherapy response, inhibiting pathways that drive drug resistance in various cancers [29]. While miR-34a is a proven tumor-suppressive miRNA with documented *in vivo* anti-cancer activity, its development as a safe and effective therapeutic for clinical use has encountered multiple challenges [30,31].

## Challenges with advancing miRNAs clinically

Nuclease susceptibility, immunogenic effects, and delivery-associated toxicity pose significant barriers to advancing miRNAs as clinical therapeutics. Unmodified miRNAs are highly susceptible to degradation by nucleases, which reduces gene-targeting efficiency and often requires high or repeated dosing to achieve therapeutic effects [32]. Although miRNAs generally elicit a lower immune response compared with plasmid DNA-based gene therapies, exogenous RNA can still activate the innate immune system, leading to the secretion of inflammatory cytokines and type I interferons through Toll-like receptors [33,34]. Beyond concerns of stability and immunogenicity, the lack of safe, specific, and effective delivery is a major hurdle, especially when targeting non-hepatic tissues [35,36]. Furthermore, for miRNA therapies to be effective, sufficient accumulation in the target cell population is essential for gene silencing; however, barriers such as the endothelial barrier, renal clearance, and endosomal entrapment significantly limit this accumulation [37,38].

The clinical development of MRX34, a formulation encompassing a miR-34a mimic with minimal chemical modifications encapsulated in a lipid nanoparticle, has provided valuable insights into the challenges with advancing miRNA therapy to patients [39,40]. The clinical trial was prematurely halted following five severe adverse events, including four fatalities. The limited clinical efficacy observed, with only 3 out of 66 evaluable patients showing a partial response, further underscores the difficulties with advancing miRNAs clinically. Although the liposomal delivery vehicle used in MRX34 has been used successfully without significant toxicity in other contexts, suggesting that it was not responsible for the adverse events, concerns regarding the immunogenic nature of the unmodified miRNA were raised. Another significant challenge was the need for repetitive and high dosing to achieve the desired therapeutic response, primarily due to the sensitivity of unmodified RNA to degradation by nucleases and lack of targeting by the delivery vehicle.

Moving forward, the successful development of miRNA therapeutics will necessitate the creation of safe delivery vehicles that efficiently deliver the miRNA to the intended organ/tissue. Indeed, biodistribution studies in non-human primates revealed high heterogeneous delivery of MRX34 across organs. Delivering therapeutic RNAs to non-hepatic tissues poses greater challenges compared with delivering to the liver, where significant progress has already been made [41]. The remainder of this mini-review will explore various strategies developed to tackle these challenges, with specific emphasis on miR-34a.

## Modified miRNAs

**Figure 1:**
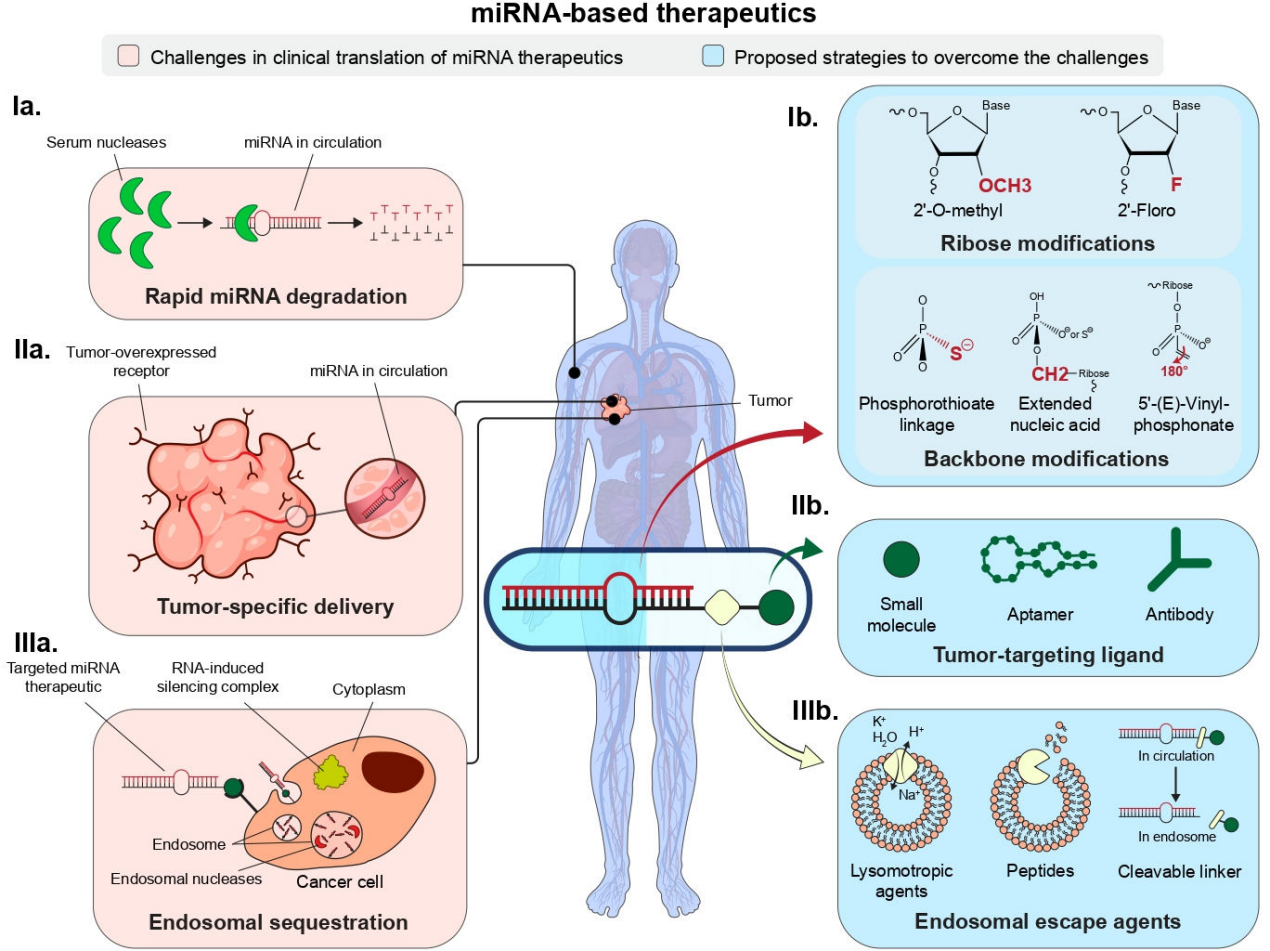
Current challenges in translating miRNA-based cancer therapeutics into the clinic and proposed strategies to overcome them. (**Ia)** Preventing rapid miRNA degradation by serum and endosomal nucleases through (**Ib**) chemical modifications of the ribose and backbone, (**IIa**) achieving tumor-specific delivery via (**IIb**) conjugation to a tumor-targeting ligand, and (**IIIa**) promoting endosomal escape through (**IIIb**) incorporation of novel chemistries.

The rational design of chemical modifications used when engineering an miRNA therapeutic is essential for enhancing stability, pharmacokinetic properties, and biological activity while simultaneously minimizing immunogenicity and off-target effects [42]. Various modification chemistries have been developed, each targeting specific sites within the RNA molecule [43,44]. Commonly used modifications include alterations to the ribose, backbone, and bases, as well as modifications at the terminal ends of the RNA, some of which are critical for ligand-mediated delivery and endosomal escape (Figure 1).

## Ribose modifications

Ribose modifications play a pivotal role in enhancing the binding affinity of RNA therapeutics to their target mRNAs and protecting them from nuclease degradation. For RNA interference (RNAi), these modifications involve substituting the highly reactive 2′-OH group of the ribose sugar with various chemical groups, such as 2′-O-methyl (2′-OMe) and 2′-fluoro (2′-F) (Figure 1Ib) [45]. Although subtle, these hydroxyl group modifications can significantly influence the ribose conformation, thereby affecting the affinity of the RNA for its targets. This underscores the need for a thorough evaluation of the modification pattern and its impact on the transcriptome.

Ribose modifications have been extensively tested in siRNA-based therapeutics to ensure optimal binding affinity, reduced immune responses, and increased target specificity, laying the foundation for similar strategies to be applied to other RNA therapeutics. Chemical modifications at the 2′ position are particularly important, as they not only improve targeting but also significantly reduce immune responses by evading recognition by pattern recognition receptors. For instance, 2′-OMe modifications are commonly used to prevent immune system activation in response to delivered RNA, a critical problem that surfaced during the MRX34 clinical trial [46]. Additionally, 2′-F modifications enhance the binding affinity of siRNAs to their target mRNAs, optimizing the siRNAs function within the RNA-induced silencing complex (RISC), making 2′-F highly suitable for RNAi-based therapeutics [47].

Efficient RISC loading requires either passenger strand cleavage or dissociation from the guide strand [48]. Since full chemical modifications enhance resistance to cleavage, dissociation or unwinding of the duplex is the only viable option to facilitate RISC loading. However, increased thermal stability between the sense and antisense strands can hinder strand separation, limiting effective RISC loading. In fact, the thermal melting temperature of a fully modified duplex is a primary factor that limits its activity. To address this, an asymmetric design in fully modified siRNAs was tested, lowering the melting temperature of the double-stranded region. This adjustment significantly improved siRNA efficacy [49]. Thus, adjustments to sense strand length can further optimize stability and functionality. Notably, the activity boost from asymmetric design is sequence-dependent. This principle is even more critical for miRNAs that contain mismatches, as they bypass cleavage and rely solely on dissociation of the two strands for RISC loading.

## Backbone modifications

The phosphorothioate (PS) backbone modification is widely used in chemically modified RNA therapeutics to enhance biological activity without significantly compromising the inherent function of the RNA. This modification replaces a non-bridging oxygen atom in the phosphate backbone with a sulfur atom, leading to changes in the hydrophilicity of the oligonucleotides that improves resistance to nuclease degradation and enhances the affinity of the RNA for serum proteins (Figure 1Ib) [50]. This increased binding to serum albumin prolongs the circulation half-life of RNA by preventing renal clearance [51]. The PS linkages also promote endocytosis of RNAs through their interaction with cell surface proteins.

Despite these advantages, PS modifications can reduce the target affinity of siRNA, leading to diminished RNAi activity [52]. To mitigate this issue, PS modifications are typically limited to the terminal ends of siRNAs, with the region analogous to the seed sequence in miRNAs left unmodified [53]. Optimizing the extent of PS modifications is crucial for balancing enhanced stability and target binding affinity. Excessive PS modifications can lead to off-target effects and nonspecific binding to certain plasma proteins, potentially altering biodistribution, sequestration, and clearance and increasing the risk of toxicity. Another challenge posed by PS modifications is the generation of stereoisomers, resulting in racemic mixtures with varying pharmacokinetic and pharmacodynamic properties, complicating clinical development [54]. Advances in stereoselective PS modification techniques, such as those pioneered by Wave Life Sciences, offer a promising path forward for improving precision and efficacy of RNA therapeutics [55].

To further enhance siRNA stability while addressing the limitations of PS modifications, researchers have explored additional backbone modifications, such as addition of a methylene group between the 5′-C and 5′-OH of a nucleoside, leading to the development of an extended nucleic acid (ExNA, Figure 1Ib). A single ExNA significantly enhances molecular stability of the RNA protecting it from 3′ and 5′ exonucleases without compromising Argonaute 2 (Ago2) loading or RNAi activity [56]. However, careful placement of ExNA is essential, as localization in the seed region of the guide strand can reduce RNAi activity. The combination of ExNA and PS modifications may also influence plasma protein binding, affecting pharmacokinetic properties and tissue distribution, which warrants further investigation.

## Targeting ligands

Vehicle-free delivery of RNAi molecules can overcome the limitations of poor specificity and cellular uptake. This can be accomplished by directly conjugating the RNA to targeting ligands, including small molecules, antibodies, aptamers, and CpG oligodeoxynucleotides (Figure 1IIb) [57]. By incorporating an azide linker on the sense strand of an siRNA or miRNA, any targeting ligand, fluorophore, or other small molecules can be conjugated to the RNA [58]. This can be achieved, for example, by synthesizing the small molecule with a Dibenzocyclooctyne (DBCO) moiety, allowing for conjugation to the azide-containing sense strand via bi-orthogonal click chemistry [58,59].

To achieve targeted RNA delivery, naturally occurring or synthetic ligands engineered to bind specific receptors that are overexpressed on cancer cells enable selective delivery of therapeutic RNAs to tumors with minimal uptake by normal cells. An ideal candidate pair that achieves these benchmarks is the folate receptor (FR) and its high-affinity ligand folic acid or folate. FR is overexpressed in various epithelial cancers, including breast, lung, ovarian, kidney, and colon cancers, osteosarcoma, as well as in certain hematological malignancies such as acute myeloid leukemia [60]. In contrast, FR expression in normal tissues is limited, providing a large therapeutic window to achieve targeted drug delivery. Folic acid or folate, the natural ligand for FR, can be easily conjugated to RNA using click chemistry without a significant loss in receptor interaction, maintaining nanomolar affinity. In addition to folate and FR, other ligand–receptor pairs have been exploited for delivery, warranting further investigation. For instance, the synthetic ligand DUPA (2-[3-(1,3-dicarboxypropyl) ureido] pentanedioic acid) was identified based on its specificity toward prostate-specific membrane antigen (PSMA), a receptor frequently up-regulated in prostate cancer [61]. This ligand was used to deliver therapeutic miR-34a, albeit with reduced efficacy compared with folate-conjugated miR-34a, demonstrating the potential use of other ligands for achieving targeted delivery of small RNAs across various cancer types [62].

While small molecules, such as folate, DUPA, and PSMA-617, are making headway for delivery of miRNAs to various tumors, aptamers, CpG oligodeoxynucleotides (ODNs), and antibodies have also been explored [63]. Aptamers are short oligonucleotides that bind to target proteins with high specificity, enabling them to deliver RNAi molecules, including miRNAs, to cancer cells [64–68]. CpG ODNs are synthetic oligonucleotides that bind to Toll-like receptor 9 (TLR9) and are rapidly internalized by cells, inducing immune responses. TLR9, which is primarily expressed by dendritic and B cells, is also up-regulated in various tumors, including prostate cancer and glioma, making CpG-ODNs a promising delivery mechanism for RNAi molecules to these cancers [69,70]. While larger than small molecules and CpG-ODNs, antibody-mediated delivery of RNAi molecules offers several advantages for RNAi delivery, including high binding affinity to their antigens [71,72]. However, their large molecular weight may lead to slow penetration of solid tumors, and their potential immune activation could affect treatment efficacy and safety. An emerging alternative is nanobodies (Nbs), the smallest known functional antibodies, measuring approximately one-tenth the size of conventional monoclonal antibodies (mAbs). Due to their compact structure, Nbs exhibit enhanced penetration into tumors, interacting more effectively with internal regions compared with mAbs or bispecific antibodies [73]. Their strong affinity, stability, and solubility make them well-suited for targeted delivery. Notably, researchers have engineered a nucleic acid nanogel for miR-34a which is targeted to cancer cells using anti-epidermal growth factor receptor (EGFR) Nbs, which led to a robust anti-tumor effect [74].

While delivery is a first step, achieving delivery at therapeutic levels is critical. High-affinity scaffold proteins, such as DARPins and Centyrins, enhance RNAi delivery by selectively binding to specific antigens, improving therapeutic outcomes [75,76]. Other approaches include lipid-conjugated RNAi molecules, particularly cholesterol-conjugates that show improved cellular uptake and bioavailability across various diseases [77–80]. However, these lipid-conjugated RNAi molecules face limitations, including lack of specificity and significant accumulation in the liver, kidney, and spleen. Optimizing lipid structures can help minimize non-specific uptake, and careful selection of RNAi molecules that only affect diseased cells is essential. While generally achievable for siRNA therapeutics, this specificity may be challenging for miRNAs due to their broad range of targets.

Combining these targeting strategies with RNAs that are chemically modified enables elimination of traditional encapsulating delivery vehicles [44]. This approach offers significant advantages, as conventional systems are often large and complex and rely on cationic lipids, which are frequently associated with toxicity [81,82]. Even newer amphoteric delivery technologies, engineered to become cationic within the tumor microenvironment, may struggle to effectively target metastatic lesions, where conditions may not be optimal for triggering the cationic state. By integrating ligand targeting with chemical modifications, encapsulating delivery vehicles can be bypassed altogether, achieving direct targeting of both primary tumors and metastatic sites, provided the target receptor is expressed. These smaller ligand-mediated delivery strategies also provide improved penetration through dense tumor architecture in challenging tumor environments.

## Endosomal escape agents

Incorporating additional chemistries is essential not only for enhancing RNA stability but also for overcoming the challenge of endosomal entrapment—a major barrier to effective RNA targeting. Efficient delivery into the cytosol is critical, as most miRNA targets reside there. However, after cellular uptake, RNA molecules are frequently sequestered in endosomes, significantly limiting their therapeutic efficacy [38]. Two main strategies used to achieve endosomal escape include: (i) inducing endosomal rupture or disruption and (ii) promoting gradual escape without rupture. Importantly, the previously discussed chemical modifications to the RNA provide stability needed for these endosomal escape mechanisms to function effectively, which often rely on increased time and harsher environments.

Small endolytic molecules can effectively mediate endosomal disruption and are often integrated into targeting chemistry (Figure 1IIIb). One such molecule that exploits the ion concentration differences between early endosomes and the cytoplasm is the ionophore nigericin. Nigericin facilitates the exchange of potassium (along with water) from the cytosol for osmotically inactive hydrogen ions in the endosome, creating an osmotic gradient that leads to endosome swelling. Through generating an intramolecular RNAi delivery vehicle that contains nigericin and a targeting ligand, such as folate, endosomal escape of an miRNA (i.e. miR-34a) or an siRNA conjugate is facilitated, increasing distribution in the cytoplasm where the RNAi is bioactive [62,83,84]. An additional strategy includes the use of peptides derived from viruses (e.g. influenza hemagglutinin HA) or insect toxins (e.g. bee venom melittin), leveraging their natural fusogenic or membrane-disrupting properties to facilitate endosomal escape [85,86]. For example, HA is a trimeric fusogenic protein that consists of an outer hydrophilic domain (HA1) that conceals an inner hydrophobic fusogenic/endosomal escape domain (HA2). Only once in the endosome, does the protein undergo conformational changes, losing the outer HA1 and incorporating the hydrophobic HA2 into the endosomal lipid bilayer, driving escape into the cytoplasm without significant toxicity. To address the rate-limiting endosomal escape problem in RNA therapeutics, novel endosomal escape domains have been designed to mimic this viral escape mechanism [87]. These peptides can significantly enhance RNAi activity by promoting cytosolic delivery and generating non-toxic byproducts at therapeutic doses. Additionally, many endosomal disruption methods unintentionally cause endosomal membrane rupture, releasing contents into the cytosol that activate the innate immune system. Thus, care must be taken to understand and develop endosomal escape chemistries that balance release with limited innate immune activation [38].

Enhancing the stability of the RNA within the nuclease-rich environment of the endosome can help with supporting gradual endosomal escape of RNA molecules. However, high-affinity interactions between the targeting ligands and receptors within the endosome can hinder effective release. To overcome this, cleavable linkers, such as disulfide bonds (S-S) or deoxythymidine linkers (dT), between the ligand and RNA have resulted in significant improvements in endosomal escape leading to benefits in gene silencing (Figure 1IIIb) [53]. These cleavable linkers are simple and effective, offering an optimal balance of stability in circulation and cleavage in the endosome compared with more complex synthetic linkers. For example, phosphodiester DNA remains intact long enough in circulation to achieve tissue distribution before being rapidly degraded following cellular uptake, allowing release of the more stable, modified RNA into the endosomal lumen, thereby increasing the chances of escape into the cytoplasm. Similar positive effects have been observed with cleavable linkers in GalNAc-conjugated siRNAs [88].

## Terminal modifications of chemically modified RNA

The 5′ phosphate group is essential for loading miRNAs into the RISC. However, when miRNAs are delivered systemically, the 5′ phosphate is often rapidly cleaved in circulation. A synthetic variant, 5′-(E)-vinylphosphonate (5′VP), provides greater resistance to phosphatases and 5′-3′ exonucleases in the bloodstream, increasing the amount of miRNA successfully loaded into RISC (Figure 1Ib) [89,90]. Additionally, studies suggest that the 5′VP forms a more energetically favorable interaction with Argonaute, enabling the modified RNA to outcompete endogenous miRNAs for Ago2 loading [91]. This competitive advantage has been shown to improve tissue retention and extend gene silencing effects *in vivo*.

## Toward the first clinically viable miRNA therapy

**Figure 2:**
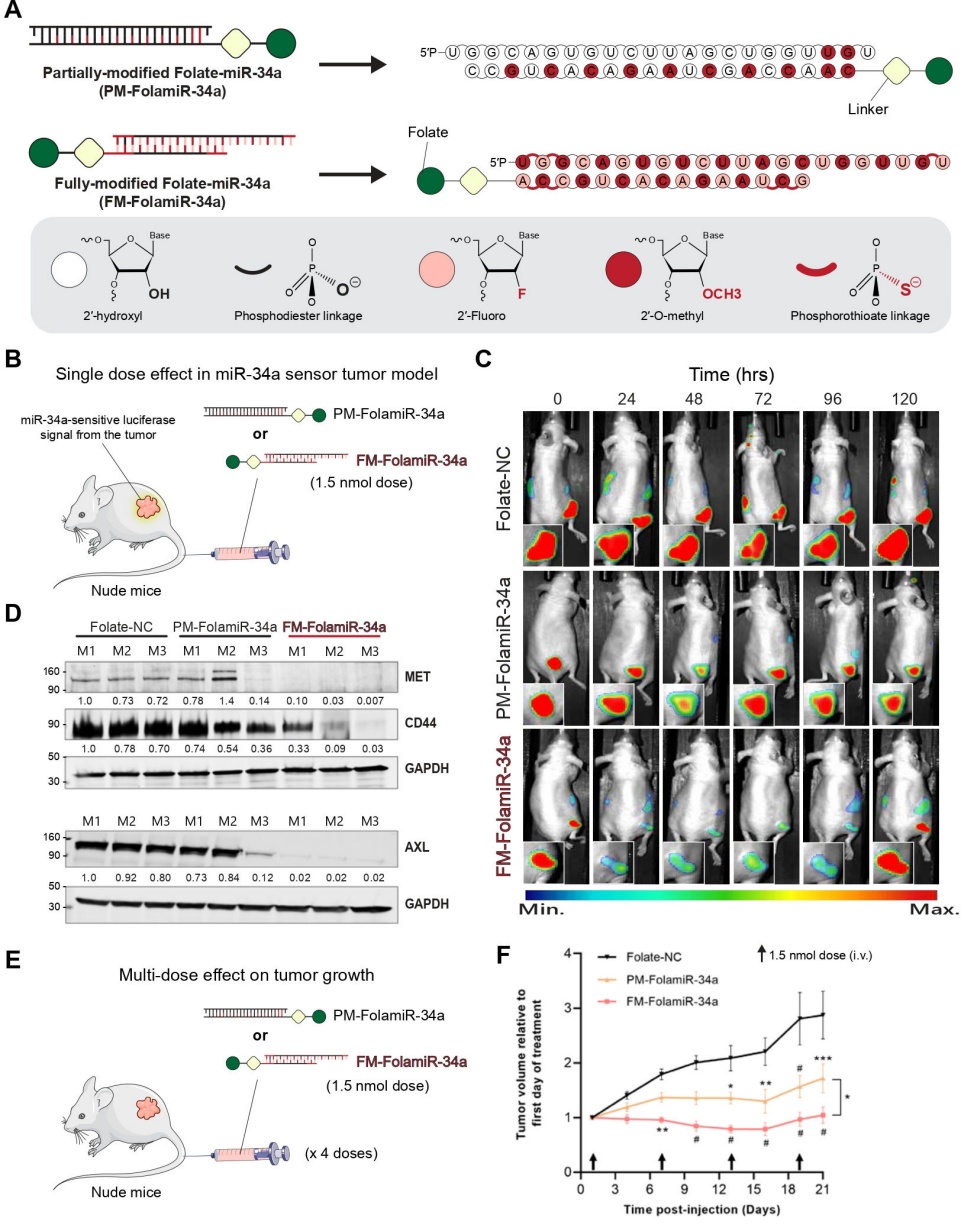
Preclinical studies using fully modified miR-34a highlight superior anti-tumor activity. (**A** *) *Chemical modification pattern of a partially modified (PM) and fully modified (FM) folate-conjugated miR-34a*.* (**B**) Schematic representation of a single dose *in vivo* study*.* (**C**) Representative images of *Renilla luciferase* sensor signal in nude mice implanted with MB-231-miR-34a sensor cells following intravenous injection of a single 1.5 nmol dose of Folate-NC, PM-FolamiR-34a, or FM-FolamiR-34a*.* (**D**) Immunoblot image of miR-34a targets (MET, CD44, and AXL) in excised MB-231 tumors 120 h after intravenous injection with a single 1.5 nmol dose of Folate-NC, PM-FolamiR-34a, or FM-FolamiR-34a duplexes, M: Mouse (n = 3 mice per treatment)*.* (**E**) Schematic representation of a multi-dose *in vivo* study. (**F**) Tumor volumes following treatment with the various Folate-miRNA conjugates. Folate-NC – Folate ligand conjugated to a non-targeting negative control (NC), PM-FolamiR-34a – Folate ligand conjugated to PM-miR-34a, FM-FolamiR-34a – Folate ligand conjugated to FM-miR-34a. Arrows represent dosing time (1.5  nmol, intravenous injection, once every 6 days) (adapted from [44,44]).

Using many of the modifications discussed in this review, the first fully modified version of miR-34a (FM-miR-34a) was developed, marking a milestone as the first fully modified miRNA with significant *in vivo* activity (Figure 2) [44]. Previously, a partially modified version of miR-34a directly conjugated to folate (FolamiR-34a) or DUPA (DUPAmiR-34a) was delivered to cancer cells, resulting in modest activity [92]. Building on this foundation, FM-miR-34a was conjugated to folate or DUPA, generating FM-FolamiR-34a and FM-DUPAmiR-34a, respectively [44,62,93]. *In vivo* delivery of FM-FolamiR-34a to mice with breast cancer xenografts led to enhanced and prolonged target gene repression, significantly outperforming the 1st generation FolamiR-34a chemistry. Critical oncogenes, such as MET, AXL, and CD44, were down-regulated by over 90% for at least 120 hours following a single 1.5 nmol dose delivered intravenously (Figure 2B–D). Collectively, FM-FolamiR-34a demonstrated increased stability, higher activity at lower doses, undetected immunogenicity in mice, and significantly reduced tumor growth leading to complete cures in two of six treated mice (Figure 2E–F). In prostate cancer xenografts, *in vivo* delivery of FM-DUPAmiR-34a significantly slowed tumor growth, with an added benefit observed when conjugated with the endosomal escape ionophore, nigericin (62).

## Future perspectives

While the enhanced targeting observed for fully modified miRNAs is exciting, it is important to consider that incorporating extensive modifications could exacerbate off-target effects. Enhanced stability and tighter binding can influence interactions with unintended sequences, potentially leading to off-target gene silencing and adverse effects [94]. As such, full transcriptomic profiling of cells exposed to unmodified and fully modified chemistries is needed to evaluate any adverse off-targeting. While nearly impossible to avoid off-targets, the on-target response should overshadow any off-target effects. In the case of FM-miR-34a, a significant portion of the on-target genes was more strongly down-regulated than those of unmodified miR-34a, albeit some off-targets were identified. Another challenge that has surfaced with the use of miRNAs as therapeutics is the potential for target-directed miRNA degradation (TDMD), which occurs with unusually strong binding between the 3′ end of the miRNA and the mRNA. This triggers degradation of the miRNA, reducing therapeutic efficiency and reliability [95]. Addressing both unintended molecular targeting and TDMD is therefore a critical aspects that needs to be assessed for each miRNA before clinical advancement.

While chemical modifications are important, not all modifications are suitable for miRNAs; the chosen modifications must balance stability with preserving biological activity. Unlike siRNAs, which bind with full complementarity to their targets, miRNAs operate based on seed sequence binding, making them particularly sensitive to modification patterns and more difficult to identify a pattern that maintains repression of critical targets while reducing off-target effects. Additionally, some modifications commonly used in other oligonucleotide chemistries are incompatible with RNAi. For example, 2′-Methoxyethyl (2′-MOE) modifications used in antisense oligonucleotides are relatively bulky and can disrupt the interaction of miRNAs with the Argonaute protein [96] leading to 2′-OMe and 2′-F being the modifications of choice for miRNAs. Bridged nucleic acids, such as locked nucleic acid (LNA), unlocked nucleic acid (UNA), and ethylene bridged nucleic acid, are generally not preferred for miRNAs due to the essential requirement of Argonaute activity, which can be disrupted with some of these modifications. An additional concern is the rigidity of consecutive LNA units within oligonucleotides, which has been linked to hepatotoxicity [97]. And, although siRNAs modified with UNA retain their on-target silencing functionality while significantly reducing off-target effects, this characteristic may pose a problem for miRNA therapeutics [98]. miRNAs have multiple target genes, and the ability to maintain on-targeting while curbing off-target silencing could inadvertently compromise the therapeutic effectiveness of miRNA-based treatments. Even subtle changes, such as 2′ ribose modifications, require careful consideration to avoid compromising miRNA function, underscoring the importance of fine-tuning modifications for therapeutic application.

Other future perspectives include increasing the circulation time of the delivered RNAs, altering the stoichiometry of ligand:miRNA to increase the intracellular dose of a single miRNA or to provide diversity through delivery of two or more relevant miRNAs, and using artificial intelligence (AI) or other platforms to inform of and develop patient-specific synthetic miRNAs. Indeed, with the advent of fully modified miRNAs, the ability to consider enhancing circulation half-life has become a reality. This can be achieved through PS modifications that lead to increased interactions of the miRNA with serum albumin or through additional chemistry that adds an albumin binding moiety to the ligand conjugate. And, while some receptors, like the folate receptor, are expressed at levels that can lead to a therapeutic level of miRNA in the cell, most receptors are not expressed at as high a level. Achieving therapeutic quantities of miRNAs in the desired cells can be accomplished through increasing the stoichiometry between the miRNA and ligand, delivering multiple miRNAs per ligand. This approach is in opposition to trivalent GalNAc conjugates that use three targeting ligands to deliver a single siRNA, an approach that, for GalNAc, helps to cluster multiple asialoglycoprotein receptors (ASGPR) to facilitate receptor internalization [99]. Finally, as basic biology is expanded and the knowledge is used and adapted to AI, the opportunity to develop synthetic miRNAs that target a cohort of genes specific to the patient/disease is becoming a reality. In the future, AI could also significantly contribute to the design and prediction of optimal RNA modifications.

While these advancements are promising, several critical factors must be addressed before clinical translation. The failure of MRX34 due to immune-related adverse events underscores the need for thorough evaluation of immune stimulation and the effectiveness of ribose modifications in mitigating unwanted immune responses. Additionally, these molecules must undergo rigorous assessment of absorption, distribution, metabolism, and excretion (ADME) to determine drug-like properties and understand how they are processed in the body.

Dosing strategies also require careful optimization—specifically, determining appropriate administration frequency. Increased stability may enable front-loading, allowing the therapeutic cargo to withstand intracellular degradation and leak from the endosome over an extended period. Finally, scaling up synthesis and formulation of modified miRNA therapeutics is essential to ensure feasibility for clinical application.

## Conclusion

In conclusion, the rational design of chemical modifications used for miRNA therapeutics is a complex process requiring a careful balance between those that affect stability, efficacy, and safety. Ongoing development and optimization of these modifications—including ribose sugar alterations, backbone modifications, phosphate stabilization, and endosomal escape strategies—are essential for advancing miRNA-based therapies. Continued research and innovation in these areas will further enhance the effectiveness and expand the therapeutic potential of miRNA-based treatments, ultimately landing miRNAs back in the clinic.

**Perspectives**

- Nuclease susceptibility, immunogenic effects, and lack of safe and effective delivery methods pose significant barriers to advancing microRNAs (miRNAs) as clinical therapeutics. The need for innovative solutions in these areas and future refinement is essential for delivering on the promise of using miRNAs as anti-cancer agents.
- The rational design of chemical modifications in miRNA therapeutics enhances stability, pharmacokinetic properties, and biological activity while minimizing immunogenicity and off-target effects. Commonly employed modifications include alterations to the ribose, backbone, bases, as well as modifications at the terminal ends of the RNA, some of which are critical for conjugating to ligands and endosomal escape agents.
- Future development of miRNA therapeutics will focus on fine-tuning chemical modifications to balance stability and biological activity, alongside creating safe and efficient delivery vehicles for targeted accumulation in cancer cells. Additional efforts need to focus on increasing circulation time and using AI to develop synthetic miRNAs, capable of targeting multiple genes specific to a particular tumor/patient.

## Abbreviations

ADME: absorption, distribution, metabolism, and excretion
AR: androgen receptor
ASGRP: asialoglycoprotein receptor
Ago2: Argonaute 2
DUPA: Dibenzocyclooctyne
EGFR: Epidermal growth factor receptor
FR: folate receptor
LNA: locked nucleic acid
2′-MOE: 2′-Methoxyethyl
Nbs: nanobodies
ODNs: oligodeoxynucleotides
PS: phosphorothioate
PSMA: prostate-specific membrane antigen
RISC: RNA-induced silencing complex
RNAi: RNA interference
TLR9: Toll-like receptor 9
UNA: unlocked nucleic acid
mAbs: monoclonal antibodies
miRNAs: MicroRNAs
